# Ethnicity corrections in pulmonary function test reports: what to do?

**DOI:** 10.1183/13993003.00571-2024

**Published:** 2024-05-02

**Authors:** Mike Hughes

**Affiliations:** National Heart and Lung Institute, Imperial College School of Medicine, Hammersmith Hospital, London, UK

## Abstract

The 2023 American Thoracic Society (ATS) document on race and ethnicity in pulmonary function test interpretation advocating “race-neutral prediction equations” [1], and the subsequent editorial on the same subject, in a recent issue of the *European Respiratory Journal* [2], are timely, in spite of some disagreement. For many years, pulmonary function laboratories have (alternatively, they may have chosen not to) reduced the predictions (based on age, height and sex) for lung volumes and capacities (but not for the transfer factor, *T*_LCO_) by 10–15% for patients of African or Asian ancestry. In my book on pulmonary function, published 13 years ago [3], I said (p. 262) “… a practical solution would be to ‘note’ the ethnic origin in the pulmonary function report, rather than correct the lung volumes by an arbitrary figure”.

## Shareable abstract

**Current opinions diverge about the need for corrections to lung volume prediction equations for people of non-European ancestry. This letter favours neither position at the present time, but recommends that “ethnic” corrections, if applied, must be stated.** https://bit.ly/4aAZwrV

*To the Editor:*

The 2023 American Thoracic Society (ATS) document on race and ethnicity in pulmonary function test interpretation advocating “race-neutral prediction equations” [1], and the subsequent editorial on the same subject, in a recent issue of the *European Respiratory Journal* [2], are timely, in spite of some disagreement. For many years, pulmonary function laboratories have (alternatively, they may have chosen not to) reduced the predictions (based on age, height and sex) for lung volumes and capacities (but not for the transfer factor, *T*_LCO_) by 10–15% for patients of African or Asian ancestry. In my book on pulmonary function, published 13 years ago [3], I said (p. 262) “… a practical solution would be to ‘note’ the ethnic origin in the pulmonary function report, rather than correct the lung volumes by an arbitrary figure”.

Over the past decade, scientific opinion, based on data, has stressed the social, genetic, environmental, neonatal (even antenatal) influences on what can be considered “healthy lungs” for diverse populations [1, 2]; the role played by ancestral or ethnic origins has been controversial, partly for lack of a plausible scientific hypothesis to promote it and partly for lack of sufficient data in populations of non-European ancestry. The ATS statement [1] recommends that such ethnicity “corrections” be replaced by a set of “universal” or “race-neutral” prediction equations, culled from as diverse a set of scientific studies as possible. The subsequent editorial in the *ERJ* [2] advocates more research before abandoning ethnicity-based corrections; the trouble is that it will take years to collect data which adequately represents the ancestral diversity of populations worldwide.

The basis for “adjustments” to volume and lung capacity predictions is the difference in ratios for lung size to standing height in populations of non-European descent [4, 5]. Although these differences exist (and will continue in the future), it should be remembered that other indices of lung function and overall respiratory “performance”, unrelated to stature, and more “efficiency” based, such as arterial oxygen tension [6], carbon dioxide tension and pH, have not been shown to have an ancestral/ethnic bias, nor is there significant sex/gender difference. For pulmonary diffusion, for example, there were no differences in the transfer coefficients (*K*_CO_ and *K*_NO_) between healthy sub-Saharan Africans and healthy European Caucasians [7], even though, for a given height, lung volumes (including the single breath alveolar volume, *V*_A_) were about 20% lower in the group with African ancestry.

The downside of removing lung volume ethnicity “corrections” is that in risk assessments based on them – for life insurance, for noninvasive ventilation support in neuromuscular weakness, for lung transplantation and lung cancer resection, for example (see table 1 in the ATS statement [1]) – patients of non-European descent may come off worse if “uncorrected” z-scores for forced expiratory volume in 1 s and forced vital capacity are applied. Since these “corrections” are based on inadequate sampling worldwide [1], there is a case for giving less weight to lung volume and spirometric measurements on their own, and paying more attention to “performance-based” indices such as maximum rate of oxygen consumption, walking distance and *K*_CO_. Note the suggestions made in table 1 in the recent editorial [2].

My view (already expressed [3]) is that it must be stated whether lung volume corrections have or have not been made. Future research will decide whether they are justified or not.

## Shareable PDF

10.1183/13993003.00571-2024.Shareable1 This one-page PDF can be shared freely online.
Shareable PDF ERJ-00571-2024.Shareable
