# Supplementary material for: Ethnicity corrections in pulmonary function test reports: what to do?
Source: Eur Respir J. 2024 May 2;63(5):2400571. doi: 10.1183/13993003.00571-2024 (PMC11063618; doi:10.1183/13993003.00571-2024)
Supplement: Supplementary file 1 [file ERJ-00571-2024.Shareable.pdf]

SHAREABLE PDF

Ethnicity corrections in pulmonary function test reports: what to do?

Mike Hughes

National Heart and Lung Institute, Imperial College School of Medicine, Hammersmith Hospital, London, UK.

Corresponding author: Mike Hughes (mike.hughes@imperial.ac.uk)

Shareable abstract (@ERSpublications)
Current opinions diverge about the need for corrections to lung volume prediction equations for people of non-European ancestry. This letter favours neither position at the present time, but recommends that "ethnic" corrections, if applied, must be stated. https://bit.ly/4aAZwrV

Cite this article as: Hughes M. Ethnicity corrections in pulmonary function test reports: what to do?. *Eur Respir J* 2024; 63: 2400571 [DOI: 10.1183/13993003.00571-2024].

This extracted version can be shared freely online.

Copyright ©The authors 2024

This version is distributed under the terms of the Creative Commons Attribution Licence 4.0.

Received: 21 March 2024
Accepted: 25 March 2024

To the Editor:

The 2023 American Thoracic Society (ATS) document on race and ethnicity in pulmonary function test interpretation advocating "race-neutral prediction equations" [1], and the subsequent editorial on the same subject, in a recent issue of the *European Respiratory Journal* [2], are timely, in spite of some disagreement. For many years, pulmonary function laboratories have (alternatively, they may have chosen not to) reduced the predictions (based on age, height and sex) for lung volumes and capacities (but not for the transfer factor, T_{LCO}) by 10–15% for patients of African or Asian ancestry. In my book on pulmonary function, published 13 years ago [3], I said (p. 262) "… a practical solution would be to 'note' the ethnic origin in the pulmonary function report, rather than correct the lung volumes by an arbitrary figure".
